# An amino acid fertilizer improves the emergent accumulator plant *Nasturtium officinale* R. Br. phytoremediation capability for cadmium-contaminated paddy soils

**DOI:** 10.3389/fpls.2022.1003743

**Published:** 2022-10-10

**Authors:** Ran Zhang, Qin Liu, Xiangting Xu, Ming’an Liao, Lijin Lin, Rongping Hu, Xian Luo, Zhihui Wang, Jin Wang, Qunxian Deng, Dong Liang, Hui Xia, Xiulan Lv, Yi Tang, Xun Wang

**Affiliations:** ^1^ College of Horticulture, Sichuan Agricultural University, Chengdu, China; ^2^ Institute of Pomology and Olericulture, Sichuan Agricultural University, Chengdu, China; ^3^ Institute of Sichuan Edible Fungi, Chengdu, China

**Keywords:** amino acid fertilizer, cadmium, *Nasturtium officinale*, phytoremediation, physiology

## Abstract

Cadmium (Cd) contamination of paddy soil affects safe crop production. This study aimed to evaluate the effects of plant biostimulant amino acid fertilizer on the phytoremediation capability of an emergent accumulator plant *Nasturtium officinale* R. Br. for Cd-contaminated paddy soils. A pot study was carried out to study the effects of different concentrations of amino acid fertilizer on the Cd accumulation of *N. officinale* grown in Cd-contaminated paddy soil. The amino acid fertilizer increased the biomass of *N. officinale*. The amino acid fertilizer concentration exhibited a quadratic polynomial regression relationship with the root and shoot biomass. The fertilizer also increased the photosynthetic pigment (chlorophyll and carotenoid) contents, peroxidase (POD; EC 1.11.1.7) activity, and catalase (CAT; EC 1.11.1.6) activity of *N. officinale*, but decreased the soluble protein content and had no significant effect on the superoxide dismutase (SOD; EC 1.15.1.1) activity. Furthermore, the amino acid fertilizer increased the Cd content and Cd extraction of *N. officinale*. The shoot Cd extraction increased by 29.06%, 63.05%, 77.22%, and 17.40% at 1500-, 1200-, 900-, and 600-fold dilutions of the amino acid fertilizer, respectively, compared with the control. Moreover, the amino acid fertilizer promoted the Cd transport from the roots to shoots of *N. officinale*. The amino acid fertilizer concentration also exhibited a quadratic polynomial regression relationship with the root Cd content, shoot Cd content, root Cd extraction, and shoot Cd extraction, respectively. The correlation, grey relational, and path analyses revealed that the root biomass, shoot biomass, chlorophyll content, catalase activity, shoot Cd content, and root Cd extraction were closely associated with the shoot Cd extraction. Therefore, the amino acid fertilizer can promote Cd uptake and improve the phytoremediation capability of *N. officinale* to remediate Cd-contaminated paddy soils, and 900-fold dilution is the most suitable concentration.

## Introduction

Soil contamination with heavy metals has become a global problem, for which an urgent solution is needed urgent solution. Heavy metals such as cadmium (Cd), copper (Cu), and arsenic (As) are often leached from contaminated farmland and mining areas, and transported into water bodies, eventually polluting paddy fields (Zhang et al., 2019; Xiao et al., 2019). Due to its high mobility, toxicity, and ease of absorption by plants, Cd can easily affect crops, posing health risks to humans through the food chain (Song et al., 2015; Zhang et al., 2020). Cd is a non-essential element in plants; therefore, its long-term absorption by crops inhibits cell division, delays growth, damages the cell structure, and disrupts metabolism, ultimately reducing crop quality and yield (Shahid et al., 2017). This necessitates the remediation of the Cd-contaminated soil.

The techniques for remediating heavy metal-contaminated soil are categorized into physical, chemical, and biological remediation methods (Shahid et al., 2017; Mahmoud et al., 2021; Wang et al., 2021b). Despite their simplicity, physical and chemical remediation methods are costly compared to bioremediation. Phytoremediation, a form of bioremediation, is cost-effective, highly efficient, and eco-friendly (Shaheen and Rinklebe, 2015). However, the phytoremediation technique is time-consuming, and the materials of phytoremediation (hyperaccumulators) have varying accumulation capabilities depending on the climatic seasons, thus limiting their application in heavy metal remediation (Grzegórska et al., 2020). Therefore, it is important to improve the phytoremediation capability of hyperaccumulators. Plant biostimulants are biologically active substances, such as amino acid, humic acid, seaweed extract, chitin, and chitosan derivatives, which can promote plant growth and improve the plant resistance to adversities (Xie et al., 2019). Among these biostimulants, amino acids can also improve nutrient utilization, increase plant biomass, enhance the uptake of trace elements, and chelate heavy metals to protect plants from heavy metal poisoning (Patrick, 2015; Xie et al., 2019). Plants respond to heavy metal stress through various cellular responses, including synthesizing metal detoxification peptides and ligand-metal complex regionalization (Qadir et al., 2014). A previous study has reported that proline, associated with synthesizing metal detoxification peptides, increases in plants when the Cd is absorbed (Sharma and Dietz, 2006). Some studies have also found that exogenous proline acid enhances Rubisco (ribulose-1,5-bisphosphate carboxylase oxygenase; EC 4.1.1.39) activity and accelerates the dark reaction stage of photosynthesis in stressful environments (Chen et al., 2004). The contents of glutathione (a plant antioxidant) have also been shown to increase in maize roots to relieve Cd stress (Wang et al., 2021a). Moreover, Khodamoradi et al. (2017) demonstrated that the application of histidine decreased the Cd content in triticale under Cd stress; however, the Cd content in the root symplast of bread wheat increased. Proline has also been shown to alleviate the harmful effects of Cd on pigeon pea (Hayat et al., 2021). Application of amino acid has been reported to increase the plant height and shoot dry weight, and the Cd absorption and extraction of the hyperaccumulator *Solanum nigrum* (Yang et al., 2009). Moreover, Richau (2009) demonstrated that histidine application increased the nickel content in the xylem of *Thlaspi caerulescens*. Thus, amino acids can enhance heavy metal absorption in hyperaccumulators to improve their phytoremediation capability for remedying the heavy metal-contaminated soil.

*Nasturtium officinale* R. Br. is a highly branching perennial herb belonging to the Cruciferae family (Zhou et al., 1987). This plant grows rapidly, with a high biomass and ability to absorb nitrogen, phosphorus, and other nutrients (Hu et al., 2011). *N. officinale* is also a Cd-accumulator that can be used to remedy the Cd-contaminated paddy fields (Lin et al., 2015). Although the Cd-phytoremediation capability of *N. officinale* is weaker than that of the terrestrial Cd-hyperaccumulators, such as *Thlaspi caerulescens*, *Sedum alfredii*, and *Viola baoshanensis* (Pence et al., 2000; Liu et al., 2003; Xiong et al., 2004), it still has the potential for improvement. The water soluble amino acid fertilizer has been widely used in crops, and is also used to improve the phytoremediation capability of hyperaccumulators (Yuan et al., 2015; He et al., 2019). So, this study aimed to determine how different concentrations of water soluble amino acid fertilizer affect the Cd uptake and phytoremediation capability of *N. officinale* in Cd-contaminated soil. Our findings provide insights into the suitable amino acid fertilizer concentration for improving the Cd accumulation of *N. officinale*, and provide a reference for remedying Cd-contaminated paddy fields.

## Materials and methods

### Materials

*N. officinale* cuttings (10 cm long shoot tips) were collected from the ditch at the Ya’an Campus of Sichuan Agricultural University (29° 59′ N, 102° 59′ E).

Soil samples were collected from the fields around the Chengdu Campus of Sichuan Agricultural University, (30°42′N, 103°51′E). The soil samples had no detectable Cd, and their physicochemical properties (**Table 1**) were the same as the report of Tang et al. (2022).

**Table 1:** The physicochemical properties of soil (Tang et al., 2022).

Soil type	pH value	Total N content (g kg^−1^)	Total P content (g kg^−1^)	Total K content (g kg^−1^)	Alkali hydrolysis N content (mg kg^−1^)	Available P content (mg kg^−1^)	Available K content (mg kg^−1^)	Total Cd content (mg kg^−1^)
Fluvo-aquic	7.09	1.50	0.76	18.02	94.82	63.30	149.59	Not detected

The water soluble amino acid fertilizer used in this experiment was produced by Shanxi Kingshine Biotechnology Co., Ltd. (China). It contained the total amino acid (various amino acids; ≥ 100 g L^−1^) and a mixture of copper + iron + manganese + zinc + boron concentration (≥ 20 g L^−1^).

### Experimental design

The experiment was conducted in a greenhouse at the Chengdu Campus of Sichuan Agricultural University. In August 2020, the soil samples were prepared as described by Lin et al. (2020), and 3.0 kg of the soils were put into each plastic pot and treated with the pure analytical Cd chloride (in the form of CdCl_2_·2.5H_2_O) to make a final soil Cd concentration of 5 mg kg^−1^, according to Tang et al. (2022). The soil was mixed immediately when the pure analytical Cd chloride was added, and was completely mixed again every 10 days. The soil was watered every day for a month to maintain in a submerged state. A month later, cuttings of *N. officinale* with uniform thickness were selected. The lower part of cuttings with 5 cm in length was cut into Cd-contamined soil, and three cuttings were planted in each pot. Because *N. officinale* grew fast, the water depth in the pots was kept at 2 cm above the soil surface during the first week after planting, and adjusted to 5 cm above the soil surface at the following weeks. One week after planting, the leaves of *N. officinale* seedlings were sprayed with the different concentrations of water soluble amino acid fertilizer (0-, 1500-, 1200-, 900-, and 600-fold dilution) until the solution started dripping from the leaves (Liao and Zhu, 2022). Each pot was sprayed 25 mL fertilizer solution. The water soluble amino acid fertilizer was sprayed again a week later after the first spraying phase. Each treatment was conducted in triplicate, and *N. officinale* seedlings were watered daily, with water depth being maintained at 5 cm above the soil surface.

### Determination of parameters

In October 2020 (thirty days after the first spraying phase), the fourth and fifth mature leaves were collected by cutting with scissors from each *N. officinale*. The leaf samples were then used for the photosynthetic pigments (chlorophyll *a*, chlorophyll *b*, and carotenoid) contents, antioxidant enzymes [peroxidase (POD; EC 1.11.1.7), superoxide dismutase (SOD; EC 1.15.1.1), and catalase (CAT; EC 1.11.1.6)] activities, and soluble protein content determinations.

The photosynthetic pigments were extracted using the acetone-ethanol extraction method, and their contents were determined according to the methods by Hao et al. (2004). Similarly, the SOD, POD, and CAT activities were determined as described by Lin et al. (2020) and Hao et al. (2004). The Coomassie brilliant blue method was adopted for the soluble protein content determination, as described by Lin et al. (2020) and Hao et al. (2004). The determination method details of photosynthetic pigment content, antioxidant enzyme activity, and soluble protein content were described in **Supplementary Material**.

Subsequently, whole plants were harvested and treated as described by Lin et al. (2020). Briefly, the plants were dried, and their dry weight biomass was weighed using an electronic balance. The dried plant tissues were then finely ground and digested as described by Lin et al. (2020) for the Cd content determination, using an ICAP6300 ICP spectrometer (Thermo Scientific, Waltham, MA, USA). Additionally, the soil samples were collected from each pot and pre-treated as described by Lin et al. (2020) for determining the soil pH value and bioavailable Cd concentration. The soil pH value was determined using a pH meter in soil-water solution (soil: water 1:2.5), while the soil bioavailable Cd concentration was determined using an ICAP6300 ICP spectrometer after extraction with DTPA-TEA (Bao, 2000). The determination method details of plant Cd content, soil pH value, and soil bioavailable Cd concentration were described in **Supplementary Material**.

The root bioconcentration factor (BCF) was estimated as the root Cd content/soil bioavailable Cd concentration; the shoot bioconcentration factor (BCF) was estimated as the shoot Cd content/soil bioavailable Cd concentration; the translocation factor (TF) was estimated as the shoot Cd content/root Cd content (Rastmanesh et al., 2010), while the Cd extraction was calculated as plant Cd content × plant biomass (Zhang et al., 2010).

### Statistical analysis

All data were analyzed using SPSS 20.0.0 software (IBM, Chicago, IL, USA). Data were normalized and subjected to a homogeneity test, followed by a one-way analysis of variance and Duncan’s Multiple Range Test (*P* < 0.05). Moreover, the quadratic polynomial regression relationship between amino acid fertilizer concentration and root biomass, shoot biomass, root Cd content, shoot Cd content, root Cd extraction, or shoot Cd extraction was analyzed using regression analysis. Pearson’s correlation was used to determine the relationships among the different indicators. The grey associations of the plant biomass, Cd content, root Cd extraction, photosynthetic pigment content, antioxidant enzyme activity, soluble protein content, soil pH value, and soil bioavailable Cd concentration with the shoot Cd extraction were evaluated *via* the grey relational analysis, according to Tang and Feng (2006); Wang (2019), and Ma et al. (2022). These associations were also subjected to path analysis, according to Tang and Feng (2006). The details of grey relational and path analyses were described in **Supplementary Material**.

## Results

### Biomass of *N. officinale*

**Figure 1:**
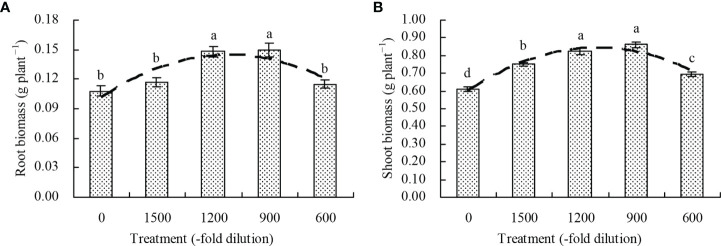
Biomass of *N. officinale*. **(A)** root biomass; **(B)** shoot biomass. Values are means ( ± SD) of three replicates. Different lowercase letters indicate significant differences among the treatments (Duncan’s Multiple Range Test, *P* < 0.05). The dashed line represents the curve of quadratic polynomial regression relationship between the amino acid fertilizer concentration and root biomass or shoot biomass.

Compared with the control, the amino acid fertilizer increased the root biomass at the 1200- and 900-fold dilution, but its concentration at the 1500- and 600-fold dilution had no significant effects (*P* > 0.05; **Figures 1A, B**). The shoot biomass increased by 22.71%, 34.80%, 40.85%, and 13.24%, respectively, at the amino acid fertilizer concentrations of 1500-, 1200-, 900-, and 600-fold dilution, respectively, compared with the control. Moreover, the regression analysis showed that the amino acid fertilizer concentration exhibited a quadratic polynomial regression relationship with both the root biomass (x: amino acid fertilizer concentration; y: root biomass; y= -4.043E^-8^x^2^ + 7.573E^-5^x + 0.103, R^2^ = 0.513, *P* = 0.013) and shoot biomass (x: amino acid fertilizer concentration; y: shoot biomass; y = -1.841E^-7^x^2^ + 0.596, R^2^ = 0.741, *P* = 0.000).

### Photosynthetic pigment content in *N. officinale* leaves

**Figure 2:**
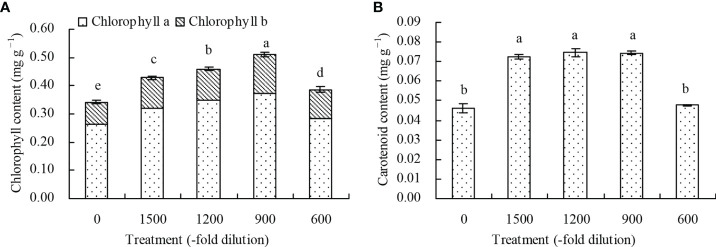
Photosynthetic pigment content in *N. officinale* leaves. **(A)** chlorophyll content; **(B)** carotenoid content. Values are means ( ± SD) of three replicates. Different lowercase letters indicate significant differences among the treatments (Duncan’s Multiple Range Test, *P* < 0.05).

The chlorophyll (chlorophyll *a* and *b*) contents in *N. officinale* leaves increased with the different concentrations of amino acid fertilizer compared with the control (**Figure 2A**). The 1500-, 1200-, 900-, and 600-fold dilutions of amino acid fertilizer increased the chlorophyll content by 25.44%, 34.21%, 49.71%, and 12.87%, respectively, compared with the control. Additionally, the carotenoid content in *N. officinale* leaves by 56.49%, 81.47%, and 61.26% at the 1500-, 1200-, and 900-fold dilutions of amino acid fertilizer, respectively, compared with the control. However, the amino acid fertilizer concentration at the 600-fold dilution had no significant effects (*P* > 0.05) on the carotenoid content (**Figure 2B**).

### Antioxidant enzyme activity and soluble protein content of *N. officinale* leaves

**Figure 3:**
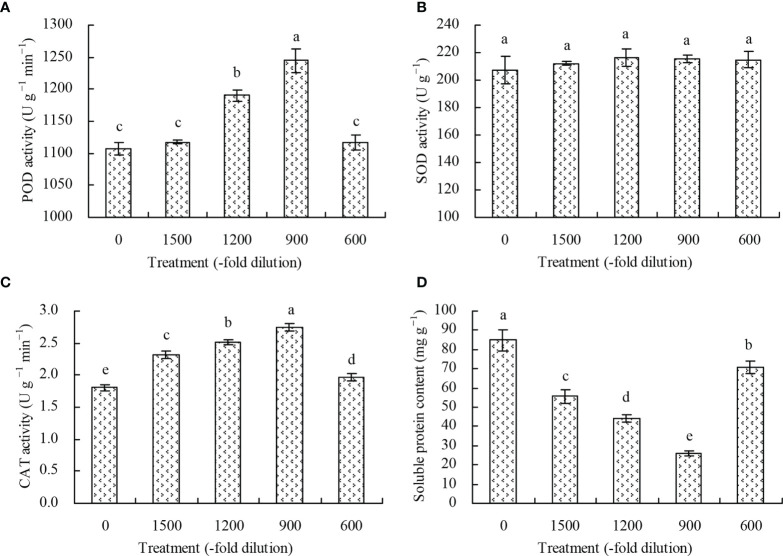
Antioxidant enzyme activity and soluble protein content of *N. officinale* leaves. **(A)** POD activity; **(B)** SOD activity; **(C)** CAT activity; **(D)** soluble protein content. Values are means ( ± SD) of three replicates. Different lowercase letters indicate significant differences among the treatments (Duncan’s Multiple Range Test, *P* < 0.05).

The POD activity of *N. officinale* leaves increased at the 1200- and 900-fold dilutions of amino acid fertilizer compared with the control, while the 600- and 1500-fold dilutions of amino acid fertilizer had no significant effects (*P* > 0.05) (**Figure 3A**). Conversely, the different concentrations of amino acid fertilizer had no significant effects (*P* > 0.05) on the SOD activity of *N. officinale* leaves (**Figure 3B**), but increased the CAT activity (**Figure 3C**). The CAT activity was the highest at the 900-fold dilution of amino acid fertilizer, followed by the 1200-, 1500-, and 600-fold dilutions, while the control had the least CAT activity. The soluble protein content in *N. officinale* leaves decreased at the different dilution folds of amino acid fertilizer, with 900-fold dilution having the least soluble protein content, followed by the 1200-, 1500-, and 600-fold dilutions. (**Figure 3D**).

### Cd content, bioconcentration, and transport of *N. officinale*

**Figure 4:**
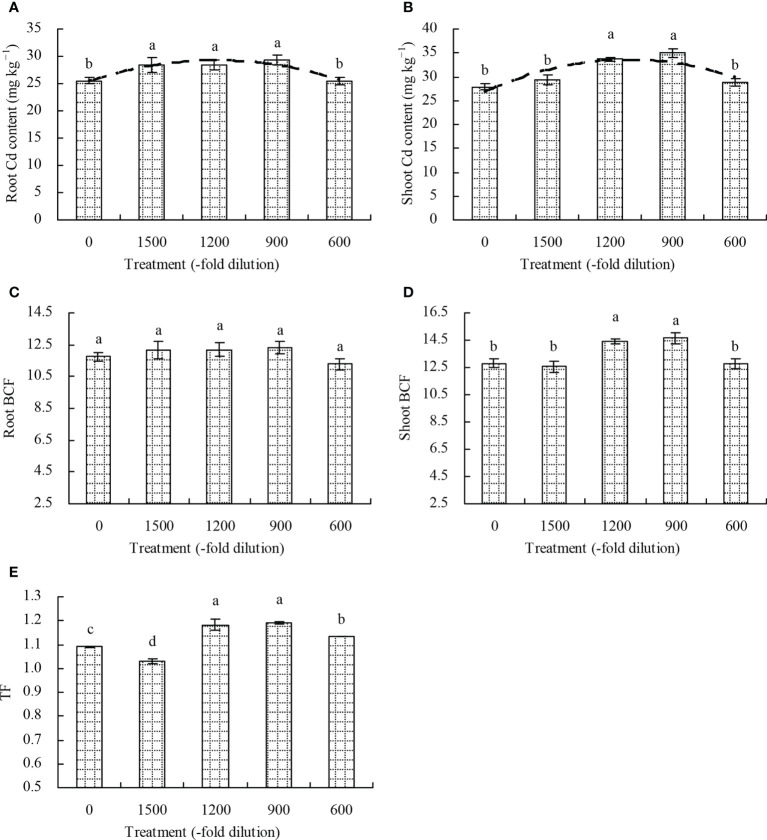
Cd content, bioconcentration, and transport of *N. officinale*. **(A)** root Cd content; **(B)** shoot Cd content; **(C)** root BCF; **(D)**: shoot BCF; **(E)** TF. Values are means ( ± SD) of three replicates. Different lowercase letters indicate significant differences among the treatments (Duncan’s Multiple Range Test, *P* < 0.05). The dashed line represents the curve of quadratic polynomial regression relationship between the amino acid fertilizer concentration and root Cd content or shoot Cd content. Root BCF (bioconcentration factor) = root Cd content/soil bioavailable Cd concentration; Shoot BCF (bioconcentration factor) = shoot Cd content/soil bioavailable Cd concentration; translocation factor (TF) = shoot Cd content/root Cd content.

The root Cd content in *N. officinale* increased only at 1500-, 1200-, and 900-fold dilutions of amino acid fertilizer compared with the control (**Figure 4A**). The 1200- and 900-fold dilutions of amino acid fertilizer increased the shoot Cd content by 20.92% and 25.81%, respectively, while the 1500- and 600-fold dilutions had no significant effects (*P* > 0.05; **Figure 4B**). Furthermore, a quadratic polynomial regression relationship existed between the amino acid fertilizer concentration and both the root Cd content (x: amino acid fertilizer concentration; y: root Cd content; y = -8.282E^-7^x^2^ + 0.004x + 25.191, R^2^ = 0.539, *P* = 0.010) and shoot Cd content (x: amino acid fertilizer concentration; y: shoot Cd content; y = -6.705E^-6^x^2^ + 0.012x + 27.078, R^2^ = 0.506, *P* = 0.014). Moreover, the amino acid fertilizer had no significant effects (*P* > 0.05) on the root BCF of *N. officinale* (**Figure 4C**). The shoot BCF of *N. officinale* increased at the 1200- and 900-fold dilutions of amino acid fertilizer, while the 1500- and 600-fold dilutions had no significant effects (*P* > 0.05) compared with the control (**Figure 4D**). The TF of *N. officinale* was increased at the 1200-, 900-, and 600-fold dilutions of amino acid fertilizer, but decreased at the 1500-fold dilution compared with the control (**Figure 4E**).

### Cd extraction by *N. officinale*

**Figure 5:**
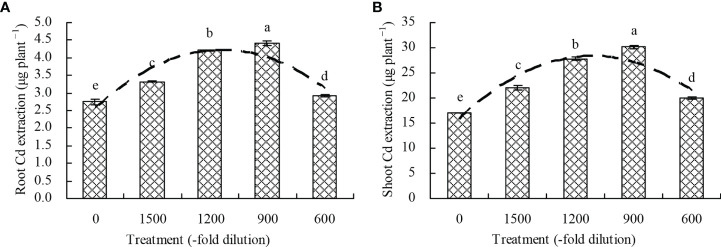
Cd extraction by *N. officinale*. **(A)** root Cd extraction; **(B)** shoot Cd extraction. Values are means ( ± SD) of three replicates. Different lowercase letters indicate significant differences among the treatments (Duncan’s Multiple Range Test, *P* < 0.05). The dashed line represents the curve of quadratic polynomial regression relationship between the amino acid fertilizer concentration and root Cd extraction or shoot Cd extraction. Root Cd extraction = root Cd content × root biomass; shoot Cd extraction = shoot Cd content × shoot biomass.

Compared with the control, the 1500-, 1200-, 900-, and 600-fold dilutions of amino acid fertilizer increased the root Cd extraction by 20.62%, 52.89%, 59.85%, and 6.10%, respectively, and also increased the shoot Cd extraction by 29.06%, 63.05%, 77.22%, and 17.40%, respectively (**Figures 5A, B**). Moreover, a quadratic polynomial regression relationship was observed between the amino acid fertilizer concentration and both the root Cd extraction (x: amino acid fertilizer concentration; y: root Cd extraction; y = -1.264E^-6^x^2^ + 0.003x + 2.581, R^2^ = 0.519, *P* = 0.012) and shoot Cd extraction (x: amino acid fertilizer concentration; y: shoot Cd extraction; y = -1.087E^-5^x^2^ + 0.021x + 15.923, R^2^ = 0.621, *P* = 0.003).

### Soil pH value and bioavailable Cd concentration

**Figure 6:**
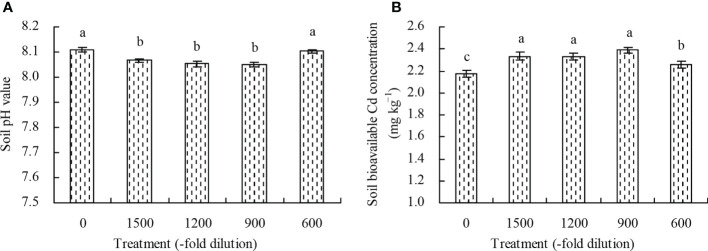
Soil pH value and soil bioavailable Cd concentration. **(A)** soil pH value; **(B)** soil bioavailable Cd concentration. Values are means ( ± SD) of three replicates. Different lowercase letters indicate significant differences among the treatments (Duncan’s Multiple Range Test, *P* < 0.05).

The 1500-, 1200-, and 900-fold dilutions of amino acid fertilizer decreased the soil pH value, while the 600-fold dilution had no significant effects (*P* > 0.05) compared with the control (**Figure 6A**). Conversely, the soil bioavailable Cd concentration increased at the different concentrations of amino acid fertilizer compared with the control (**Figure 6B**).

### Correlation analysis

The root Cd content, shoot Cd content, root Cd extraction, and shoot Cd extraction had a significant (*P* < 0.01) positive correlation with the root biomass, shoot biomass, chlorophyll content, carotenoid content, POD activity, CAT activity, and soil bioavailable Cd concentration, while exhibited a significantly (*P* < 0.01) negative correlation with the soluble protein content and soil pH value (**Table 2**). The shoot Cd content and shoot Cd extraction positively correlated (0.01 ≤ *P* < 0.05) with the SOD activity, unlike the root Cd content and root Cd extraction (*P* > 0.05). There was also a positive correlation between the shoot Cd extraction (*P* < 0.01) and the root Cd extraction. The root Cd extraction and shoot Cd extraction were positively correlated (*P* < 0.01) with the root Cd content and shoot Cd content. Contrarily, the soil bioavailable Cd concentration negatively correlated (*P* < 0.01) with the soil pH value.

**Table 2:** Correlations among biomass, plant Cd content, plant Cd extraction, photosynthetic pigment content, antioxidant enzyme activity, soluble protein content, soil pH value, and soil bioavailable Cd concentration.

Indicator	Root biomass	Shoot biomass	Chlorophyll content	Carotenoid content	POD activity	SOD activity	CAT activity	Soluble protein content	Root Cd content	Shoot Cd content	Root Cd extraction	Shoot Cd extraction	Soil bioavailable Cd concentration	Soil pH value
Root biomass														
Shoot biomass	0.934**													
Chlorophyll content	0.897**	0.981**												
Carotenoid content	0.781**	0.899**	0.884**											
POD activity	0.950**	0.887**	0.901**	0.703**										
SOD activity	0.452	0.528*	0.488	0.411	0.41									
CAT activity	0.893**	0.971**	0.984**	0.928**	0.888**	0.506								
Soluble protein content	-0.895**	-0.975**	-0.990**	-0.882**	-0.904**	-0.572*	-0.984**							
Root Cd content	0.701**	0.835**	0.874**	0.937**	0.701**	0.453	0.923**	-0.876**						
Shoot Cd content	0.936**	0.908**	0.919**	0.775**	0.942**	0.545*	0.924**	-0.922**	0.817**					
Root Cd extraction	0.976**	0.962**	0.949**	0.873**	0.944**	0.484	0.961**	-0.949**	0.839**	0.968**				
Shoot Cd extraction	0.961**	0.977**	0.974**	0.855**	0.944**	0.537*	0.970**	-0.972**	0.842**	0.976**	0.989**			
Soil bioavailable Cd concentration	0.773**	0.916**	0.907**	0.862**	0.745**	0.600*	0.907**	-0.926**	0.798**	0.754**	0.826**	0.854**		
Soil pH value	-0.859**	-0.933**	-0.909**	-0.941**	-0.783**	-0.352	-0.925**	0.897**	-0.847**	-0.806**	-0.905**	-0.891**	-0.856**	

N = 15. **: Correlation is significant at the 0.01 level (2-tailed test). *: Correlation is significant at the 0.05 level (2-tailed test).

### Grey relational and path analyses

**Figure 7:**
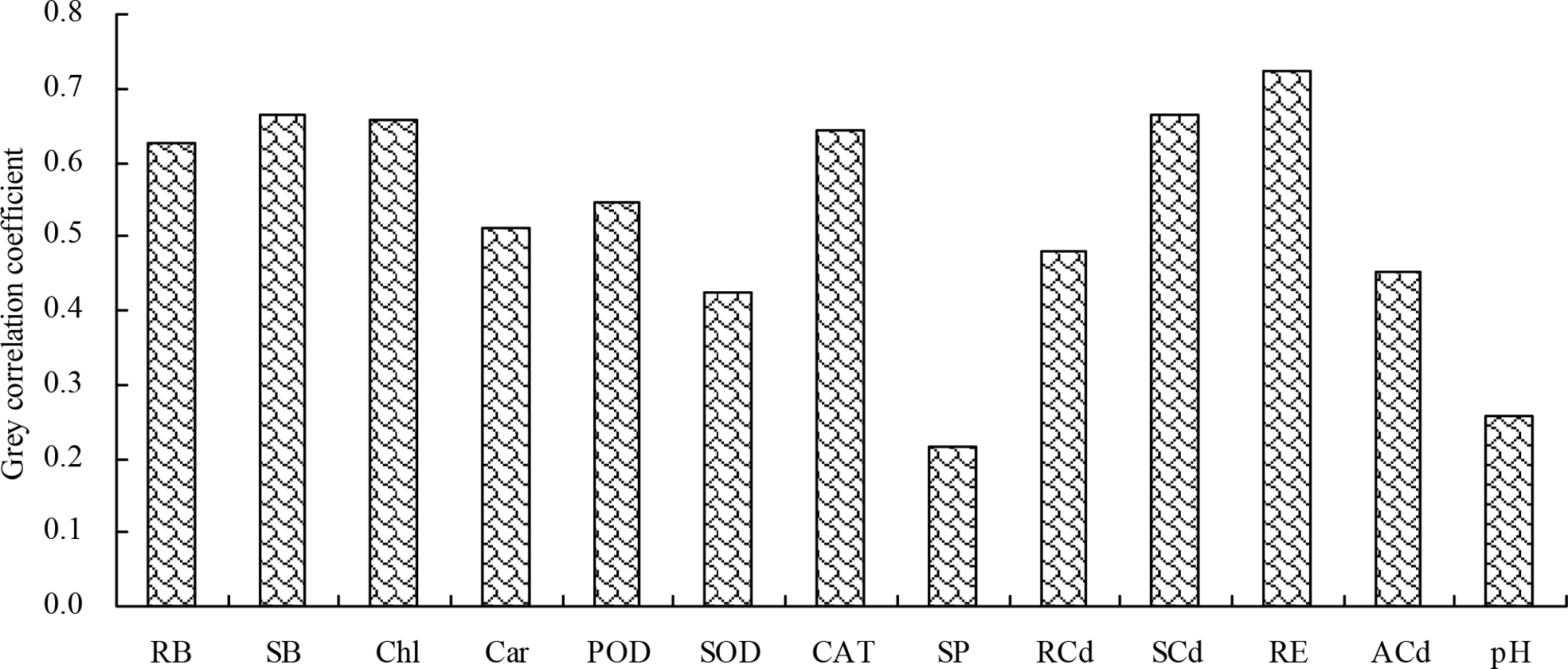
Grey correlation coefficients of the biomass, plant Cd content, root Cd extraction, photosynthetic pigment content, antioxidant enzyme activity, soluble protein content, soil pH value, and soil bioavailable Cd concentration with the shoot Cd extraction. RB, root biomass; SB, shoot biomass; Chl, chlorophyll content; Car, carotenoid content; POD, POD activity; SOD, SOD activity; CAT, CAT activity; SP, soluble protein content; RCd, root Cd content; SCd, shoot Cd content; RE, root Cd extraction; ACd, soil bioavailable Cd concentration; pH, soil pH value.

The grey relational analysis showed that the shoot Cd extraction correlated with the biomass, plant Cd content, root Cd extraction, photosynthetic pigment content, antioxidant enzyme activity, soluble protein content, soil pH value, and soil bioavailable Cd concentration (**Figure 7**). Among these indicators, the root biomass, shoot biomass, chlorophyll content, CAT activity, shoot Cd content, and root Cd extraction had grey correlation coefficient values higher than 0.60, with the shoot Cd extraction. Therefore, the root biomass, shoot biomass, chlorophyll content, CAT activity, shoot Cd content, and root Cd extraction were closely associated with the shoot Cd extraction.

**Figure 8:**
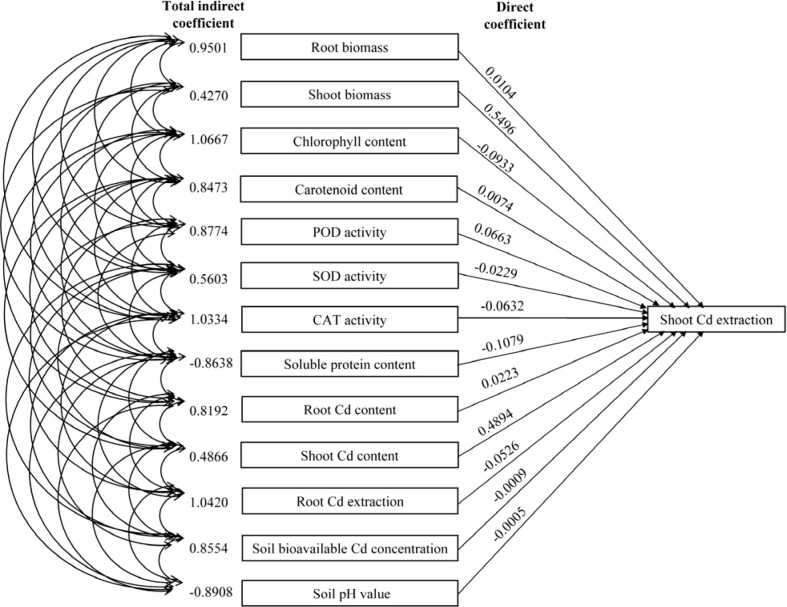
Path coefficients of the biomass, plant Cd content, root Cd extraction, photosynthetic pigment content, antioxidant enzyme activity, soluble protein content, soil pH value, and soil bioavailable Cd concentration with the shoot Cd extraction.

The direct path coefficients of the shoot biomass and shoot Cd content were higher than 0.45 compared to the path coefficient values (absolute) of the other indicators. This indicates that the shoot biomass and shoot Cd content directly affected the shoot Cd extraction (**Figure 8**; **Table S1**). Moreover, the indirect path coefficients of root biomass, chlorophyll content, CAT activity, and root Cd extraction were higher than 0.95, indicating their indirect positive effects on the shoot Cd extraction.

## Discussion

Amino acids contain carbon, nitrogen, and other elements, which provide nutrients for plants and serve as the carbon skeleton for energy metabolism in the human body (Song et al., 2012). The application of amino acids has been reported to promote the growth and increase the biomass of lettuce under hydroponic conditions (Khan et al., 2019). Similarly, an amino acid liquid fertilizer (made of pig hairs) also increased the yield of cowpea (Wang et al., 2019). Therefore, the application of amino acids promotes plant growth and improves crop yield. In this study, the amino acid fertilizer increased the biomass of *N. officinale*, consistent with previous reports by Khan et al. (2019) and Wang et al. (2019). This indicates that amino acid fertilizer could promote the growth of *N. officinale* by enhancing the synthesis of hormones in plants, thereby promoting the nutrient absorption. Amino acids also induce the expression of certain transporters, making it easier for plants to absorb and utilize nutrients (Sharma and Dietz, 2006). The amino acid fertilizer used in this experiment contained copper, iron, manganese, zinc, and boron, which could have also promoted the growth of *N. officinale*.

The application of amino acids can also promote the chlorophyll synthesis, thereby increasing the chlorophyll content and improving the photosynthetic efficiency of crops (Yao et al., 2021). Cheng et al. (2012) reported that amino acid foliar fertilizer increased the chlorophyll content of strawberries, thus improving its net photosynthetic rate and photochemical energy conversion efficiency. The addition of exogenous proline also increased the chlorophyll *a* and chlorophyll *b* contents in pigeon pea under Cd stress (Hayat et al., 2021). In this study, the amino acid fertilizer increased the chlorophyll and carotenoid contents in *N. officinale*. Under Cd stress, the Cd ions inhibit the chlorophyll synthase by replacing the central magnesium ions in chlorophylls, thereby reducing the magnesium absorptive capacity of plants (Jamers et al., 2009; Willows, 2019). Thus, the amino acid fertilizer may have reduced the inhibitory effect of Cd on chlorophyll synthase (Yao et al., 2021), thereby increasing the photosynthetic pigment content in *N. officinale*.

Under heavy metal stress, ROS are generated in plants, causing damage to plant DNA, proteins and lipids (Hernández-Jiménez et al., 2002; Orhan et al., 2004). The SOD, POD, and CAT are the main cellular enzymes that protect plants against the ROS damage (Hernández-Jiménez et al., 2002; Orhan et al., 2004; Zhang et al., 2021). A previous study has shown that amino acids increased the activity of antioxidant enzymes in soybean (Teixeira et al., 2017). Proline, a multifunctional molecule involved in ROS tolerance and scavenging, increased the activity of antioxidant enzymes in pigeon pea under Cd stress (Hayat et al., 2021). In this experiment, the amino acid fertilizer increased the POD and CAT activities of *N. officinale*, thus improving its resistance to Cd stress. However, the amino acid fertilizer had no significant effect on the SOD activity of *N. officinale*, suggesting that the SOD of *N. officinale* was not sensitive to the amino acid fertilizer. The amino acid fertilizer decreased the soluble protein content in *N. officinale*, possibly because the proteins chelated with Cd (Zhang, 2007), thus, further improving the resistance of *N. officinale* to Cd.

The soil pH value is an important factor affecting the migration and availability, adsorption-desorption, dissolution-precipitation, and other reactions of heavy metal ions in soil (Yu et al., 2016). At higher pH values (pH > 6.0), the soil Cd exists in the form of insoluble compounds resulting from complexation, chelation, and precipitation, which reduces its availability in the soil. However, at lower pH values, the complexed forms of Cd are easily converted into the bioavailable Cd, increasing the bioavailable Cd in the soil (Dou et al., 2020). In this study, the amino acid fertilizer decreased the soil pH value, thereby increasing the soil bioavailable Cd concentration. Therefore, the soil bioavailable Cd concentration negatively correlated with the soil pH value, and this increased the absorption of soil Cd by *N. officinale*. The decrease in soil pH may have been due to the secretion of organic acids by the roots of *N. officinale*, induced by the amino acid fertilizer, which increased the soil bioavailable Cd concentration (Montiel-Rozas et al., 2016). Thus, the amino acids indirectly correlate with the soil Cd concentration and the Cd accumulation in plants. High histidine contents in plants grown in nickel-containing medium improved their tolerance to nickel stress, indicating that histidine may be related to plant resistance against nickel stress (Kramer et al., 1996; Kerkeb and Kramer, 2003). Moreover, aspartic acid can chelate the Cd, lead, and zinc, reducing their toxicity (Bottari and Festa, 1996). Cysteine synthesis also improved the Cd tolerance in *Arabidopsis* and tobacco (Dominguez-Solis et al., 2004; Ning et al., 2010). In this study, the amino acid fertilizer increased the Cd content, shoot BCF, TF, and Cd extraction of *N. officinale*. These results correlate with the increased soil bioavailable Cd concentration, suggesting that amino acid fertilizer could improve the Cd phytoremediation capability of *N. officinale*. Furthermore, correlation analysis showed that the Cd contents and Cd extractions of roots and shoots positively correlated with the root biomass, shoot biomass, chlorophyll content, carotenoid content, POD activity, CAT activity, and soil bioavailable Cd concentration, and negatively correlated with the soluble protein content and soil pH value. The root and shoot Cd extractions had positive correlations with the root and shoot Cd contents. These results suggest that the amino acid fertilizer improves the phytoremediation capability of *N. officinale* by enhancing its physiological resistance and promoting its growth. Additionally, the grey relational analysis showed that the root biomass, shoot biomass, chlorophyll content, CAT activity, shoot Cd content, and root Cd extraction were closely associated with the shoot Cd extraction. The path analysis further demonstrated that the shoot biomass and shoot Cd content had the direct effects on the shoot Cd extraction, while the root biomass, chlorophyll content, CAT activity, and root Cd extraction had the indirect effects on the shoot Cd extraction.

## Conclusion

The amino acid fertilizer increased the biomass, photosynthetic pigment content, POD activity, and CAT activity of *N. officinale*, but decreased the soluble protein content, thus promoting *N. officinale* growth under Cd-contaminated soil. The amino acid fertilizer also increased the Cd content and Cd extraction of *N. officinale*, and promoted the Cd transport from the roots to shoots. A quadratic polynomial regression relationship existed between the of amino acid fertilizer concentration and the rootbiomass, shoot biomass, root Cd content, shoot Cd content, root Cd extraction, and shoot Cd extraction, respectively. Notably, the root biomass, shoot biomass, chlorophyll content, CAT activity, shoot Cd content, and root Cd extraction were closely associated with the shoot Cd extraction. Thus, the amino acid fertilizer could improve the phytoremediation capability of *N. officinale* to remediate the Cd-contaminated paddy soils, and 900-fold dilution was the most suitable concentration. Replicating this experiment in different settings would help validate the potential of amino acid fertilizer in enhancing the phytoremediation efficiency of Cd-contaminated paddy soils by *N. officinale*.

## Data availability statement

The raw data supporting the conclusions of this article will be made available by the authors, without undue reservation.

## Author contributions

RZ, QL, and XX: Investigation, Data curation, Writing- Original draft preparation. M’aL, RH, XL, ZW, JW, QD, DL, HX, XLL, YT, and XW: Investigation, Data curation. LL: Conceptualization, Methodology. All authors contributed to the article and approved the submitted version.

## Conflict of interest

The authors declare that the research was conducted in the absence of any commercial or financial relationships that could be construed as a potential conflict of interest.

## Publisher’s note

All claims expressed in this article are solely those of the authors and do not necessarily represent those of their affiliated organizations, or those of the publisher, the editors and the reviewers. Any product that may be evaluated in this article, or claim that may be made by its manufacturer, is not guaranteed or endorsed by the publisher.
