# Supplementary material for: An amino acid fertilizer improves the emergent accumulator plant Nasturtium officinale R. Br. phytoremediation capability for cadmium-contaminated paddy soils
Source: Front Plant Sci. 2022 Oct 10;13:1003743. doi: 10.3389/fpls.2022.1003743 (PMC9592069; doi:10.3389/fpls.2022.1003743)
Supplement: Supplementary file 1 [file Table_1.doc]

**Supplementary Material**

**Determination of photosynthetic pigment content**

The third mature leaves were collected and cut into pieces (about 1cm in length). Fresh samples (0.2 g) of leaf pieces were soaked in 10 mL ethanol and acetone (1:1, v/v) in the dark for 24 h, and then, the extraction solutions used to determine the absorbance values at 663, 645, and 470 nm by a spectrophotometer. The absorbance values were used to calculate the contents of chlorophyll *a*, chlorophyll *b*, and carotenoid according to Hao et al. (2004).

$$\text{C}_{a}=12.21A_{663}-2.81A_{646}$$

$$\text{C}_{b}=20.13A_{646}-5.03A_{663}$$

$$\text{C}_{Car}=(1000A_{470}-3.27\text{C}_{a}-104\text{C}_{b})/229$$

In these formulas, $\text{C}_{a}$was the chlorophyll *a* content; $\text{C}_{b}$ was the chlorophyll *b* content; $\text{C}_{Car}$was the carotenoid content; $A_{663}$was the absorbance value of chlorophyll *a*; $A_{646}$was the absorbance value of chlorophyll *b*; $A_{470}$was the absorbance value of carotenoid.

**Determination of antioxidant enzyme activity and soluble protein content content**

The fourth mature leaves were collected and homogenized in 6 mL extraction buffer [0.05 M potassium phosphate buffer (pH 7.0) containing 1 mM EDTA] at 4°C, and the homogenate was centrifuged at 11,000 ×*g* for 20 min at 4°C. The supernatant was collected and used for analysis of antioxidant enzyme (SOD, POD, and CAT) activity and soluble protein content. According to the methods of Lin et al. (2020) and Hao et al. (2004), the SOD activity was determined by the nitro blue tetrazolium reduction method, the POD activity was determined by the guaiacol method, the CAT activity was determined by the hydrogen peroxide method, and the soluble protein content was determined by the coomassie brilliant blue G-250 method.

**Determination of plant Cd content**

The dried plant tissue samples were finely ground for a chemical analysis. Plant samples (0.5 g) were soaked in 25 mL HNO_3_-HClO_4_ (4:1 vol/vol) for 12 h, and digested at a heating plate with 200 °C until the solution became transparent. After that, the volume of solution was increased to 50 mL with deionized water and filtered. The Cd concentration of filtered solution was determined with the iCAP 6300 ICP-MS spectrometer (Thermo Scientific, Waltham, MA, USA). Then, the root Cd and shoot Cd contents were calculated using the Cd concentration of filtered solution according to Lin et al. (2020). The Cd measurements were checked with certified standard reference material (GBW-07602; bush branches and leaves) obtained from the China National Center for Standard Reference Materials according to Lin et al. (2020).

**Determination of soil pH value and soil bioavailable Cd concentration**

The pot soils were collected, air-dried, and sieved through a 1-mm mesh for determining the soil pH value and soil bioavailable Cd concentration. The soil samples (10 g) were added 25 mL deionized water (soil:water 1:2.5). After the soil solution was stirred completely and stood for 30 min, the soil pH value was measured with a pH meter according to Bao (2000). The soil samples (5 g) were added 25 mL 0.005 mol/L diethylenetriaminepentaacetic acid (DTPA) and triethanolamine (TEA) for extracting 2 h in a shaker. The extraction solution was filtered, and the Cd concentration of filtered solution was determined with the iCAP 6300 ICP-MS spectrometer (Thermo Scientific, Waltham, MA, USA). The soil bioavailable Cd concentration was calculated using the Cd concentration of filtered solution according to Bao (2000).

**Grey relational analysis**

The grey relational analysis method is used for the quantitative comparative analysis of the degree of multi-factor correlation. In this study, the shoot Cd extraction by *N. officinale* was used as the reference sequence (X*_0_*), and the other indexes including the biomass, plant Cd content, root Cd extraction, photosynthetic pigment content, antioxidant enzyme activity, soluble protein content, soil pH value, and soil bioavailable Cd concentration [X*_i_* (*i*=1, 2, 3……)] were used as the comparison sequence. The analysis steps were three steps: (1) the data were subjected the dimensionless quantification using the normalized transformation. The mean and standard deviation of each series were found, and then, using the original data subtracted the mean and next divided by the standard deviation. The obtained new data was the normalized data. (2) The correlation coefficient was calculated using the following formula.

$$\text{L}_{\text{0i}}\text{(k) = } \frac{\Delta\min+\rho\Delta\max}{\Delta_{0i}(k)+\rho\Delta\max}$$

In the formula, was the correlation coefficient between the k-th reference data column and the comparison data column; $\Delta_{0i}(k)$ the absolute difference at point *k*, $\Delta\min$expressed as the smallest difference between the X*_0_* and X*_i_* sequence; $\Delta\max$ was the maximum difference between the sequence of X*_0_* and sequence of X*_i_*, $\rho$ represent whitening value of gray number, , and 0.1 was taken in this study. (3) The degree of gray correlation calculated using the following formula (Tang and Feng, 2006).

$$\text{r}_{\text{0i}}\text{ = }\frac{1}{n} \sum_{k=1}^{n} L_{0i}(k)$$

In the formula, was the gray correlation degree of the comparison sequence X*_i_* to the reference sequence X*_0_*, or called the sequence correlation degree. The closer the value of $\text{r}_{\text{0i}}$ was to 1, the better the correlation. The other details were described by Wang (2019) and Ma et al. (2022).

**Path analysis**

Path analysis can be used to analyze the linear relationship between multiple independent variables and dependent variables. It is an extension of regression analysis and can handle more complex variable relationships. On the basis of correlation analysis and regression analysis, the quantitative relationship (path coefficient) between the dependent variable and the independent variable can be further studied, and the correlation coefficient can be decomposed into direct effect coefficient and indirect effect coefficient to the relative importance of reveal each factor to dependent variable. The path coefficient starts from a simple correlation coefficient matrix, and then obtains the direct path coefficient and the indirect path coefficient by solving the normalized equation of the path coefficient. Assuming that in *ρ* independent variables (x_1_, x_2_, ... , x*_p_*), the simple correlation coefficient between each two variables and the dependent variable (*y*) can form a standardized equation for solving the path coefficient:

*r*_11_*ρ*_1_ + *r*_12_*ρ*_2_ + … + *r*_1_*_p_ρ_p_* = *r*_1_*_y_*

*r*_21_*ρ*_1_ + *r*_22_*ρ*_2_ + … + *r*_2_*_p_ρ_p_* = *r*_2_*_y_*

… … … … …

*r_p_*_1_*ρ*_1_ + *r_p_*_2_*ρ*_2_ + … + *r_pp_ρ_p_* = *r_py_*

In the formula, *r* is the correlation coefficient, and *ρ* is the direct path coefficient. The direct path coefficient can be obtained by calculating the inverse of the above-mentioned correlation matrix. Assuming that *C_ij_* is the inverse of the correlation matrix *r_ij_*, then the direct path coefficient *ρ* is:

$$\left(\begin{matrix} \rho_{1} \\ \rho_{2} \\ \cdots\\ \rho_{p} \end{matrix}\right)=\left(\begin{matrix} C_{11} & C_{12} & \cdots& C_{1p} \\ C_{21} & C_{22} & \cdots& C_{2p} \\ \cdots& \cdots& \cdots& \cdots\\ C_{p1} & C_{p1} & \cdots& C_{pp} \end{matrix}\right)\times\left(\begin{matrix} r_{1y} \\ r_{2y} \\ \cdots\\ r_{py} \end{matrix}\right)$$

The direct path coefficient *ρ_i_* represents as *ρ_i→y_*. The indirect path coefficient *ρ_i→j→y_* = *r_ij_ρ_j→y_* (Tang and Feng, 2006).

In this study, the shoot Cd extraction by *N. officinale* was used as the dependent variable (*y*), and the other indexes including the biomass, plant Cd content, root Cd extraction, photosynthetic pigment content, antioxidant enzyme activity, soluble protein content, soil pH value, and soil bioavailable Cd concentration were used as the independent variables (x_1_, x_2_, ... , x*_p_*).

**TABLE S1 Path coefficients of the biomass, plant Cd content, root Cd extraction, photosynthetic pigment content, antioxidant enzyme activity, soluble protein content, soil pH value, and soil bioavailable Cd concentration with the shoot Cd extraction.**

| **Factor** | **Direct effect** | **Indirect effect** | | | | | | | | | | | | | |
| --- | --- | --- | --- | --- | --- | --- | --- | --- | --- | --- | --- | --- | --- | --- | --- |
|  |  | **Total** | **X1→Y** | **X2→Y** | **X3→Y** | **X4→Y** | **X5→Y** | **X6→Y** | **X7→Y** | **X8→Y** | **X9→Y** | **X10→Y** | **X11→Y** | **X12→Y** | **X13→Y** |
| X1 | 0.0104 | 0.9501 |  | 0.5132 | -0.0836 | 0.0058 | 0.0630 | -0.0104 | -0.0565 | 0.0966 | 0.0156 | 0.4581 | -0.0514 | -0.0007 | 0.0004 |
| X2 | 0.5496 | 0.4270 | 0.0097 |  | -0.0915 | 0.0066 | 0.0588 | -0.0121 | -0.0614 | 0.1052 | 0.0186 | 0.4442 | -0.0506 | -0.0009 | 0.0004 |
| X3 | -0.0933 | 1.0667 | 0.0093 | 0.5391 |  | 0.0065 | 0.0597 | -0.0112 | -0.0622 | 0.1069 | 0.0194 | 0.4496 | -0.0499 | -0.0009 | 0.0004 |
| X4 | 0.0074 | 0.8473 | 0.0081 | 0.4942 | -0.0824 |  | 0.0466 | -0.0094 | -0.0587 | 0.0952 | 0.0209 | 0.3791 | -0.0459 | -0.0008 | 0.0004 |
| X5 | 0.0663 | 0.8774 | 0.0099 | 0.4875 | -0.0840 | 0.0052 |  | -0.0094 | -0.0562 | 0.0975 | 0.0156 | 0.4613 | -0.0497 | -0.0007 | 0.0004 |
| X6 | -0.0229 | 0.5603 | 0.0047 | 0.2901 | -0.0456 | 0.0030 | 0.0272 |  | -0.0320 | 0.0617 | 0.0101 | 0.2669 | -0.0254 | -0.0006 | 0.0002 |
| X7 | -0.0632 | 1.0334 | 0.0093 | 0.5338 | -0.0918 | 0.0068 | 0.0589 | -0.0116 |  | 0.1062 | 0.0206 | 0.4523 | -0.0506 | -0.0009 | 0.0004 |
| X8 | -0.1079 | -0.8638 | -0.0093 | -0.5357 | 0.0924 | -0.0065 | -0.0599 | 0.0131 | 0.0622 |  | -0.0195 | -0.4511 | 0.0500 | 0.0009 | -0.0004 |
| X9 | 0.0223 | 0.8192 | 0.0073 | 0.4592 | -0.0815 | 0.0069 | 0.0465 | -0.0104 | -0.0584 | 0.0945 |  | 0.3996 | -0.0441 | -0.0008 | 0.0004 |
| X10 | 0.4894 | 0.4866 | 0.0097 | 0.4989 | -0.0857 | 0.0057 | 0.0625 | -0.0125 | -0.0585 | 0.0995 | 0.0182 |  | -0.0509 | -0.0007 | 0.0004 |
| X11 | -0.0526 | 1.0420 | 0.0102 | 0.5287 | -0.0885 | 0.0064 | 0.0626 | -0.0111 | -0.0608 | 0.1025 | 0.0187 | 0.4737 |  | -0.0008 | 0.0004 |
| X12 | -0.0009 | 0.8554 | 0.0080 | 0.5035 | -0.0846 | 0.0063 | 0.0494 | -0.0137 | -0.0573 | 0.1000 | 0.0178 | 0.3690 | -0.0434 |  | 0.0004 |
| X13 | -0.0005 | -0.8908 | -0.0089 | -0.5126 | 0.0848 | -0.0069 | -0.0519 | 0.0081 | 0.0585 | -0.0968 | -0.0189 | -0.3946 | 0.0476 | 0.0008 |  |

Coefficient of determination = 1.0000; Residual path coefficient = 0.0003. X1 = root biomass; X2 = shoot biomass; X3 = chlorophyll content; X4 = carotenoid content; X5 = POD activity; X6 = SOD activity; X7 = CAT activity; X8 = soluble protein content; X9 = root Cd content; X10 = shoot Cd content; X11 = root Cd extraction; X12 = soil bioavailable Cd concentration; X13 = soil pH value; Y = shoot Cd extraction.
